# 肺腺癌、鳞癌中CD147与MMP-2的表达及意义

**DOI:** 10.3779/j.issn.1009-3419.2011.09.02

**Published:** 2011-09-20

**Authors:** 斯闻 王, 波 李, 斯阳 王, 玉 李, 军 李

**Affiliations:** 1 110005 沈阳，中国医科大学附属第四医院ICU Department of ICU, the 4^th^ Affiliated Hospital, China Medical University, Shenyang 110005, China; 2 110001 沈阳，中国医科大学附属第一医院胸外科 Department of Thoracic Surgery, the 1^st^ Affiliated Hospital, China Medical University, Shenyang 110001, China; 3 110001 沈阳，中国医科大学附属第一医院感染科 Department of Infectious Diseases, the 1^st^ Affiliated Hospital, China Medical University, Shenyang 110001, China; 4 330029 南昌，江西省肿瘤医院胸部肿瘤外科 Department of Thoracic Surgery, JiangXi Cancer Hospital, Nanchang 330029, China

**Keywords:** CD147, MMP-2, 肺肿瘤, 免疫组化, CD147, MMP-2, Lung neoplasms, Immunohistochemistry

## Abstract

**背景与目的:**

CD147是细胞外基质金属蛋白酶诱导因子，目前研究表明其与肿瘤的侵袭与转移有密切关系。本研究旨在探讨CD147和MMP-2在肺腺癌、鳞癌中的表达及其与临床病理特征的关系。

**方法:**

应用免疫组织化学方法检测55例人肺腺癌、鳞癌及其相应癌旁正常组织中CD147和MMP-2的蛋白表达，同时分析其与临床病理因素之间的关系。

**结果:**

CD147和MMP-2的蛋白表达在人肺腺癌、鳞癌组织中阳性率均明显高于癌旁肺组织。肺腺癌、鳞癌中CD147和MMP-2蛋白表达与年龄、性别及病理组织学分型无明显关系（*P* > 0.05），但与淋巴结转移及TNM分期有关（*P* < 0.05）。肺腺癌、鳞癌中CD147蛋白表达与MMP-2蛋白表达呈正相关。

**结论:**

CD147表达可能与肺腺癌、鳞癌侵袭和转移密切相关，可作为判断肺腺癌、鳞癌细胞生物学行为的客观参考指标。

## Abstract (en)

**Background and objective:**

It has been proven that CD147 was an extracellular matrix metalloproteinase inducer reportedly involved in the invasion and metastasis of malignancies. The aim of this study is to investigate CD147 and MMP-2 expression in squamous cell carcinoma and adenocarcinoma of the lungs and to analyze their clinical significance.

**Methods:**

Tissue samples from 55 patients with squamous cell carcinoma and adenocarcinoma of the lungs and their corresponding non-cancerous tissues were examined for CD147 and MMP-2 expression using immunohistochemistry.

**Results:**

The positive expression rates of CD147 and MMP-2 in the squamous cell carcinoma and adenocarcinoma among the lung tissues were significantly higher than those in the corresponding normal lung tissues. Moreover, the CD147 and MMP-2 expression in squamous cell carcinoma and adenocarcinoma of the lungs were related to lymph node metastasis and TNM stages (*P* < 0.05), but not to age, gender and histologic type (*P* > 0.05). MMP-2 expression was highly correlated with CD147 expression.

**Conclusion:**

CD147 and MMP-2 expression is correlated with the invasion and metastasis of squamous cell carcinoma and adenocarcinoma of the lungs and may be used as objective markers for predicting the behavior of squamous cell carcinoma and adenocarcinoma of the lungs.

CD147又被称为细胞外基质金属蛋白酶诱导物（extracellular matrix metalloproteinase inducer, EMMPRIN），是免疫球蛋白超家族成员之一。CD147的表达及其与CD147功能相关的蛋白质之间的作用在很多疾病中起着重要的作用，可刺激成纤维细胞大量分泌基质金属蛋白酶（matrix metalbproteinases, MMPs）刺激因子，参与降解细胞外基质，并与肿瘤侵袭转移关系密切^[1-3]^。MMP-2是MMPs超家族成员之一，它能特异性降解基质膜中的主要成分Ⅳ型胶原，使基底膜丧失完整性，还能通过毛细血管内生、新生血管生成等促进肿瘤生长和扩散^[4, 5]^。本研究采用免疫组化方法检测CD147与MMP-2在55例肺腺癌、鳞癌组织中的表达情况，并分析其与临床病理因素之间的相关性，探讨其表达状态与预测肺腺癌、鳞癌生物学行为的关系。

## 1 材料与方法

### 1.1 病例资料与标本来源

2005年3月-2005年12月在中国医科大学附属第一医院胸外科行手术治疗并经病理确诊的肺腺癌、鳞癌组织标本及其相应癌旁正常组织标本各55例。患者男34例，女21例，年龄25岁-81岁，中位年龄57.3岁。术前均未行放疗、化疗等辅助治疗，肿瘤均为单发。55例肺癌患者均行肺癌完全性切除，包括全肺切除4例，肺叶切除51例，其中切除上中叶3例，中下肺叶11例，单肺叶切除37例。根据Naruke肺癌淋巴结分布图^[6]^行系统性纵隔淋巴结廓清，55例共清扫淋巴结341组，平均每例清扫6.2组。病理组织学分型依据世界卫生组织（World Health Organization, WHO）1999年发布的上皮性肿瘤分类标准分为鳞癌31例和腺癌24例。术后病理分期根据1997年国际抗癌联盟TNM分期标准，包括I期28例、Ⅱ期9例、Ⅲ期18例。

### 1.2 主要试剂

柠檬酸型抗原修复缓冲液（MVS-0066，粉剂）购自福建迈新公司，MMP-2鼠抗人单克隆抗体（sc-13595，浓缩型）购自美国Santa Cruz公司，CD147兔抗人多克隆抗体（ZA-0455，工作液）及SP-9000通用型免疫组化试剂盒均购自北京中杉金桥生物技术公司。

### 1.3 方法

标本常规固定、脱水、石蜡包埋，4 μm切片。将柠檬酸型抗原修复缓冲液按说明书稀释成工作液（pH=6.0），将切片于修复液中高压修复1 min，SP法进行免疫组化染色，以PBS代替一抗作阴性对照。即用型CD147兔抗人多克隆抗体和MMP-2鼠抗人单克隆抗体（工作浓度1:150）4 ℃孵育过夜，DAB显色。CD147阳性反应颗粒定位于细胞膜及细胞浆，评分标准按免疫组化半定量评分法依据染色强度及阳性细胞所占百分比进行评分^[7]^。每张切片阳性细胞的染色强度按无、弱和强分别记为1分、2分、3分；按着色阳性细胞所占百分比分别记为0分、1分、2分、3分和4分（0: < 10%; 1: ≥10%- < 25%; 2: ≥25%- < 50%; 3: ≥50%- < 75%；4: ≥75%），然后根据两项记分之积判定其结果，分数 < 2分为阴性，≥2分为阳性。

### 1.4 统计学处理

采用SPSS 13.0软件进行数据处理，采用χ^2^检验、*Spearman*等级相关检验、*Kaplan-Meier*法等进行统计分析。*P* < 0.05为差异有统计学意义。

## 2 结果

### 2.1 CD147表达情况

CD147蛋白在正常肺组织中不表达（图 1A），在61.8%（34/55）的肺腺癌、鳞癌组织中呈阳性表达（图 1B，图 1C），两者表达差异有统计学意义（*P* < 0.05）。肺腺癌、鳞癌中CD147蛋白表达与年龄、性别及病理组织学分型无关（*P* > 0.05），与淋巴结转移及TNM分期有关（*P* < 0.05）（表 1）。

**1:**
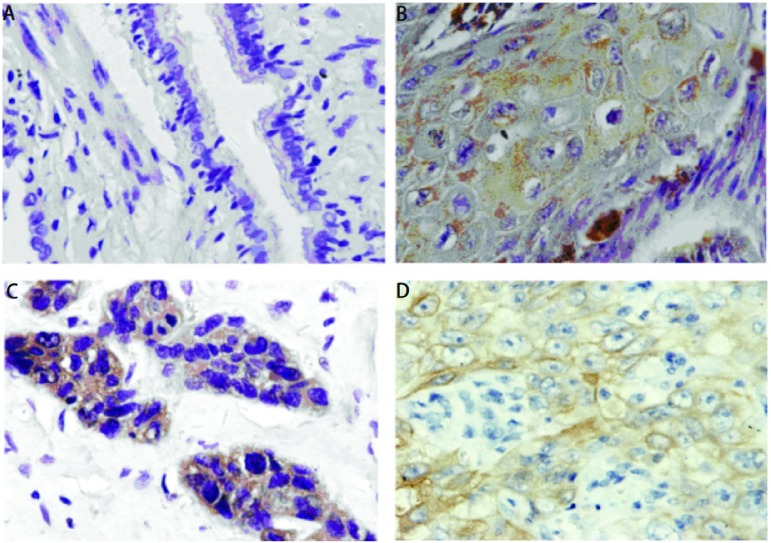
CD147在正常肺组织与原发肺鳞癌、腺癌中的表达及MMP-2在肺腺癌中的表达（SP，×400）。A：CD147在正常肺组织中无表达；B：CD147在肺鳞癌中表达于细胞膜及细胞浆；C：CD147在肺腺癌中表达于细胞膜及细胞浆；D：MMP-2在肺腺癌中表达于细胞浆。 The expression of CD147 in normal lung tissue, squamous cell carcinoma and adenocarcinoma of the lungs and the expresssion of MMP in lung adenocarcinoma (SP, ×400). A: Negative expression of CD147 in normal lung tissue; B: Positive expression of CD147 in the membrane and cytoplasm of lung squamous cell cancer cells; C: Positive expression of CD147 in the membrane and cytoplasm of lung adenocarcinoma cells; D: Positive expression of MMP in the membrane of lung adenocarcinoma cells.

**1:** 肺腺癌、鳞癌中CD147和MMP-2蛋白的表达与临床病理因素的关系 Relationship between CD147, MMP-2 expression and clinicopathological factors in squamous cell carcinoma and adenocarcinoma of the lungs

Group	*n*	CD147			MMP-2		
		Positive (*n*=34)	Negative (*n*=21)		Positive (*n*=20)	Negative (*n*=35)	
Age (yr)				*χ*^2^=2.56 *P* > 0.05			*χ*^2^=1.52 *P* > 0.05
≤55	19	9	10		9	10	
> 55	36	25	11		11	25	
Gender				*χ*^2^=1.28 *P* > 0.05			*χ*^2^=3.77 *P* > 0.05
Male	34	23	11		9	25	
Female	21	11	10		11	10	
Histological type				*χ*^2^=0.42 *P* > 0.05			*χ*^2^=2.37 *P* > 0.05
Squamous cell carcinoma	24	16	8		6	18	
Adenocarcinoma	31	18	13		14	17	
TNM stage				*χ*^2^=8.31 *P* < 0.01			*χ*^2^=7.91 *P* < 0.01
Ⅰ+Ⅱ	37	18	19		17	20	
Ⅲ	18	16	2		3	15	
Lymph node metastasis				*χ*^2^=5.72 *P* < 0.05			*χ*^2^=10.64 *P* < 0.01
Yes	27	21	6		4	23	
No	28	13	15		16	12	

### 2.2 MMP-2表达情况

MMP-2阳性染色定位于细胞浆（图 1D）。MMP-2在63.6%（35/55）的肺腺癌、鳞癌中阳性表达，在14.5%（8/55）相应癌旁正常组织中表达，差异有统计学意义（*P* < 0.05）。肺腺癌、鳞癌中MMP-2蛋白表达与年龄、性别及病理组织学分型无关（*P* > 0.05），与淋巴结转移及TNM分期有关（*P* < 0.05）（表 1）。

### 2.3 肺腺癌、鳞癌中CD147与MMP-2蛋白表达的相关性分析

在CD147阳性表达的肺腺癌、鳞癌病例中MMP-2蛋白表达阳性率为85.3%（29/34），在CD147阴性表达的肺腺癌、鳞癌病例中MMP-2蛋白表达阳性率为28.6%（6/21）。相关性分析显示肺腺癌、鳞癌中CD147与MMP-2蛋白表达呈正相关（*r*=0.573, *P* < 0.01）（表 2）。

**2:** 肺腺癌、鳞癌中CD147蛋白表达与MMP-2蛋白表达的相关性 Correlation of CD147 expression with MMP-2 expression in squamous cell carcinoma and adenocarcinoma of the lungs

	CD147 expression		Total	
	+	-		
MMP-2 expression (+)	29	6	35	*r*=0.573 *P* < 0.01
(-)	5	15	20	
Total	34	21	55	

### 2.4 CD147与MMP-2蛋白表达与肺腺癌、鳞癌预后的关系

31例鳞癌与24例腺癌从手术日开始随访5年，其中4例鳞癌与2例腺癌失访。CD147阳性表达和阴性表达5年生存率分别为38.7%和66.7%，MMP-2阳性表达和阴性表达5年生存率分别为26.7%和58.2%，生存曲线见图 2。CD147阳性表达的肺腺癌、鳞癌患者生存率低于CD147阴性表达患者（χ^2^=4.76, *P* < 0.05）；MMP-2阳性表达的肺腺癌、鳞癌患者生存率低于MMP-2阴性表达患者（χ^2^=4.31, *P* < 0.05）。

**2:**
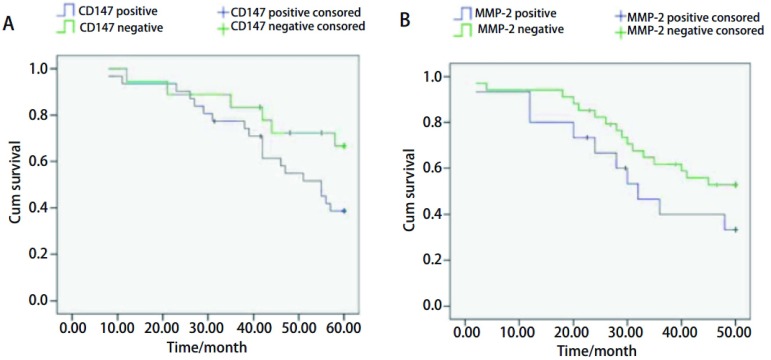
CD147与MMP-2蛋白表达与肺腺癌、鳞癌预后的关系。A：CD147阳性表达和阴性表达组肺腺癌、鳞癌患者的*Kaplan-Meier*生存曲线（*P* < 0.05）；B：MMP-2阳性表达和阴性表达组肺腺癌、鳞癌患者的*Kaplan-Meier*生存曲线（*P* < 0.05）。 The relationship between CD147, MMP-2 expression and the prognosis of squamous cell carcinoma and adenocarcinoma of the lungs. A: *Kaplan-Meier* survival curves of lung cancer patients with CD147 expression (*P* < 0.05); B: *Kaplan-Meier* survival curves of lung cancer patients with MMP-2 expression (*P* < 0.05).

## 3 讨论

肿瘤的侵袭、转移是一个多因素参与、多步骤的复杂过程，包括原发肿瘤浸润、基底膜降解、肿瘤细胞进出血管和肿瘤细胞对靶组织的侵袭等环节^[8]^。这个过程中细胞外基质起着关键性作用，它是肿瘤迁移过程中所遇到的自然屏障。MMP-2是MMPs超家族成员之一，它能特异性降解基质膜中的主要成分Ⅳ型胶原，使基底膜丧失完整性，还能通过毛细血管内生、新生血管生成等促进肿瘤生长和扩散。CD147是近年来新发现的一种可刺激成纤维细胞大量分泌MMPs的刺激因子。MMPs降解细胞外基质成分，从而为肿瘤的浸润和转移创造有利条件。CD147属于免疫球蛋白超家族，分子量约58 kDa，为单链的跨膜糖蛋白，由269个氨基酸残基组成，其胞膜外区有2个免疫球蛋白超家族结构域，这种结构域与免疫球蛋白可变区和Ⅱ类主要组织相容性抗原复合物的β链非常相似^[9]^。目前研究表明在血液系统、消化系统、泌尿系统和神经系统等均存在CD147的低表达，参与机体生殖、免疫和神经活性等重要生理过程。近年关于CD147与肿瘤关系的研究^[2, 10-14]^相继报道，在前列腺癌、结直肠癌、子宫内膜癌等多种肿瘤细胞中均发现有CD147表达的增加，部分肿瘤中还发现随着肿瘤恶性程度的增加CD147的表达也增加，且与肿瘤的浸润和转移相关。

本研究发现CD147与MMP-2在肺腺癌、鳞癌中的表达明显高于癌旁正常组织（*P* < 0.05）。CD147和MMP-2的表达均与年龄、性别及病理组织学分型无关（*P* > 0.05），而与淋巴结转移及TNM分期相关（*P* < 0.05），提示CD147与MMP-2是与肺腺癌、鳞癌恶性生物学行为关系密切的预测因子。Ⅲ期肺腺癌、鳞癌患者CD147的阳性表达率（88.9%）明显高于Ⅱ期患者（48.6%）（*P* < 0.05），有淋巴结转移的患者CD147阳性表达率（77.8%）明显高于无淋巴结转移者（46.4%）（*P* < 0.05）。本研究的结果与文献报道相符，Sun等^[15]^认为CD147通过刺激成纤维细胞及肿瘤细胞产生多种MMPs降解基底膜和细胞外基质，首先促进肿瘤的局部浸润而实现转移。本研究发现肺腺癌、鳞癌中CD147与MMP-2呈现高频率的同时阳性表达，相关性分析示肺腺癌、鳞癌中CD147蛋白表达与MMP-2蛋白表达呈正相关，提示CD147可能刺激MMPs的分泌并上调MMP-2的表达水平。Sun等^[15]^将人乳腺癌细胞系MDA-435 CD147沉默后，MMP-2表达下降且细胞侵袭性下降，认为CD147通过MMP-2在肿瘤侵袭、转移中起重要作用。Zhong等^[16]^研究发现前列腺癌中CD147表达与MMP-1, 2, 9的表达呈明显正相关，CD147和MMP-2表达与TNM及Gleason分期呈正相关，且同时表达CD147和MMP-2的患者预后差，这表明CD147与MMP-2在前列腺癌的浸润转移中起重要作用。Wu等^[18]^报道CD147与MMP-2在胆囊癌中表达明显高于慢性胆囊炎性病变，且CD147与MMP-2同时阳性表达预后最差，可见两者在胆囊的发生发展中起重要作用，联合检测可以提示胆囊癌预后。因此，CD147与MMP-2同时阳性表达是多种恶性肿瘤预后差的重要标志。Norgauer等^[18]^提出CD147刺激MMPs分泌的机制为CD147刺激肿瘤周围成纤维细胞分泌MMPs，从而导致基底膜和间质成分降解，同时能与MMPs形成复合物而加强MMPs对肿瘤间质的降解，更利于肿瘤向周围浸润及转移。

CD147在促进肿瘤细胞的浸润和转移中的作用与通过刺激肿瘤细胞和基质细胞分泌和活化MMPs有关，其主要是间质成纤维细胞分泌，包括介导肿瘤细胞之间、肿瘤细胞和基质细胞之间的相互作用，形成肿瘤细胞-细胞外基质金属蛋白酶类-基质成分构成的肿瘤侵袭、转移的局部微环境。本实验结果提示CD147和MMP-2的表达与肺腺癌、鳞癌的发生发展有关，与其侵袭、转移密切相关，两者联合检测可作为判断肺腺癌、鳞癌细胞生物学行为的客观参考指标。
